# Understanding social oocyte freezing in Italy: a scoping survey on university female students’ awareness and attitudes

**DOI:** 10.1186/s40504-019-0092-7

**Published:** 2019-05-03

**Authors:** Pamela Tozzo, Antonio Fassina, Patrizia Nespeca, Gloria Spigarolo, Luciana Caenazzo

**Affiliations:** Department of Molecular Medicine, University of Padova, via Falloppio 50, 35121 Padova, Italy

**Keywords:** Social oocyte freezing, Survey, Questionnaire, Public awareness, Oocyte donation

## Abstract

In Western countries, a social trend toward delaying childbearing has been observed in women of reproductive age for the last two decades. This delay is due to different factors related to lifestyle, such as the development of a professional career or the absence of the right partner. As a consequence, women who defer childbearing may find themselves affected by age-related infertility when they decide to conceive. Fertility preservation techniques are, therefore, proposed as a solution for these women. Among all possible solutions, social freezing is an alternative strongly discussed from a scientific, social and ethical point of view.

A survey among 930 female students at the University of Padova (Italy) investigated their knowledge and attitudes on social egg freezing and their potential intentions regarding this procedure. To our knowledge, this is the first study to examine the level of awareness of age-related infertility in Italian young women and their attitudes regarding acceptable indications for elective oocyte freezing, their potential personal use, the circumstances in which they would then decide to use cryopreserved eggs, and their attitudes towards cost coverage and oocyte donation.

Data collected in this study revealed some important points about young women and their knowledge about social oocyte freezing in Italy as compared to other European countries and the United States.

Overall, 34.3% of the students reported having heard about the possibility of oocyte cryopreservation for non-medical reasons and being aware of the meaning of this procedure; only 19.5% were in favour of social egg freezing and 48.4% thought that the cost for this procedure should be borne entirely by the woman herself. Regarding egg donation, the majority of students (64.9%) would not accept donating their eggs to a known woman or couple and 42.5% would instead accept donating to a biobank.

Our study shows that young Italian women are significantly less aware of age-related decline in fertility and the possibility of using social egg freezing compared to their similarly situated counterparts in other Western countries.

## Introduction

Over the last 30 years, in Western countries, a progressive social trend toward delaying childbearing has been observed in women of reproductive age. This delay is due to different factors related to lifestyle and societal changes, such as improved educational and professional opportunities for women, familial care committments, economic difficulties and the need for greater financial security, absence of a partner, necessity of establishing a stable home environment, improved access to contraception or a feeling of not being “ready” for parenthood. As a consequence, these women may find themselves affected by age-related infertility when they decide to conceive, and fertility preservation techniques can be proposed to them as a solution. The fact that female fecundity gradually decreases with age has been recognised by several demographic and epidemiological studies that have demonstrated a decline in fertility beginning as early as the middle of the third decade (Crawford and Steiner, 2015; Stoop et al. 2014) due to reduced oocyte quantity and quality. Such deterioration of the ovarian function, along with the trend of deferring motherhood, has resulted in an increased number both of women who remain involuntarily childless and women over the age of 45 using donor eggs rather than their own.

Primarily, egg freezing has been offered only for medical reasons to women, facing cancer treatments or other fertility-impairing conditions, who had no other options for fertility preservation. This medical innovation is now being widely promoted and offered also to healthy, fertile women who wish to postpone motherhood. While the option of freezing oocytes of cancer patients or other patients with decreased fertility is generally positively considered both from a medical and an ethical point of view, offering the same option to healthy women, for the reasons previously indicated, is met with new ethical challenges that have recently been debated in the literature (Borovecky et al., 2018; Bracewell-Milnes et al. 2018; Jones et al. 2018).

Social egg freezing means preserving and storing a woman’s oocytes for non-medical purposes and the trend toward social freezing has likely continued to increase with more and more centres providing this service to women who wish to preserve their gametes for deferred childbearing. Famous companies (Apple and Facebook, for example) have also started to offer egg-freezing benefits to female employees as a nudge policy to encourage people to make healthier choices (Ismaili M'hamdi et al. 2017; Zoll et al. 2015).

Public reaction to the meaning of this opportunity has been mixed: some viewed the development positively as a forward-thinking practice that would give greater flexibility and peace of mind to young female employees to avoid pressure because of their declining ovarian reserve, while others have been sceptical that women would be the true beneficiaries, arguing that it would create implicit pressure to partake in egg freezing and delaying motherhood in order to demonstrate seriousness and dedication to the workplace (Bracewell-Milnes et al. 2018, Jones et al. 2018).

The procedure of oocyte cryopreservation finds its completion in the application of Assisted Reproductive Technology (ART), given that the collection and storage of oocytes can be justified from the perspective of their use at a later date by the opportunity to resort to ART. In fact, elective oocyte freezing consists of two separate steps that are clearly distinct in time: first, the oocyte banking through ovarian stimulation, oocyte retrieval, cryopreservation and storage; and second, even several years later, the thawing and fertilization of the cryopreserved oocytes. At the time of the first step, women who request social freezing are healthy individuals who are asking for a procedure that results in storing their oocytes that may or may not be used in the future, depending on the further course of their lives.

When the procedure of oocyte cryopreservation is not specifically regulated, the referring normative has to be found in the Assisted Reproductive Technology, given that the collection and storage of oocytes can be justified from the perspective of their use at a later date by an appeal to ART.

In Italy, the recourse to ART is allowed only in order to assist in the resolving of reproductive problems arising as a result of human sterility or infertility (Art. 1 of Law. N. 40/2004) (Italian Parliament, 2004). Art.4 and Art.5 of the same law state that access to reproductive technologies is allowed for couples that have been medically certified as infertile/sterile whether for known or unknown causes. Furthermore, in Italy, there are two principles on which the ART can be performed: firstly, on the gradual application of the techniques, according to how invasive the procedures are and the psychological impact they have; and secondly, only to medically certified as infertile/sterile couples. Given that the demand for social egg freezing comes from healthy single women, and it is not possible to scale the application of the ART techniques, currently in Italy, from a legal point of view, it does not seem to be possible, for a single healthy woman, to access ART directly after social egg freezing. In fact, social egg freezing is possible only in private centres, outside the national healthcare system, with costs entirely covered by the woman who requests it.

Recent studies in the literature indicate a significant success of the oocyte vitrification technique to make it stable through very rapid cooling to about -100°C, where molecular activity stops (Potdar et al. 2014).

In the case of assisted reproductive techniques, the pregnancy rate per embryo transfer for women receiving in-vitro fertilization (IVF) treatment using their own fresh eggs drops, between the ages of 35 and 45, from 38.2% to 2.2%. If a woman freezes her eggs before her fertility starts to decline, IVF using her own frozen eggs will be more likely to work into her late 30s and 40s. For women freezing their eggs in their mid-20s to mid-30s, there is a clinical pregnancy rate per thawed oocyte between 4.5% and 12%. Although not identical, pregnancy rates for IVF using frozen oocytes are now broadly comparable with pregnancy rates using fresh oocytes, so that a woman who froze her eggs at the age of 35 could benefit from an IVF success rate closer to 38% than 2% into her 40s. The optimum time to freeze a woman’s eggs, from a clinical point of view, would be during a woman’s 20s also because, if a woman freezes her eggs in her late 30s, the process may be more invasive and expensive, if more cycles are needed, and the pregnancy rate per frozen oocyte will be lower, even if she is more likely to return to use her frozen eggs (Jackson 2016); nevertheless, despite this, most women come to vitrify at ages 37–39 years (Baldwin et al. 2015; Cobo et al. 2016).

From a medical point of view, we have to consider the balance between the risks of the procedures (ovarian hyper stimulation, oocyte pick up and pregnancy) and the benefits, for the mother and the child. As every medical intervention entails risks, in this case the risks are twofold, both for the mother and for the future child. For the mother many health risks are due to the In Vitro Fertilization by Intracytoplasmic Sperm Injection (IVF-ICSI) treatment, especially in woman over 45 years of age: with increasing age, pre-pregnancy chronic medical conditions and obstetrical risks and adverse birth outcomes rise; even though maternal mortality rates are very low in Europe, they are increasing with increasing age. Other concerns are the possibility of creating high and potentially false hopes and introducing medical processes to primary fertile women. Furthermore, we should also consider the risks for the future child: due to advanced maternal age and pregnancy complications, neonatal complications are also increased, comprising prematurity and lower mean birth weights among infants of older women compared with those of younger women.

The term “elective freezing”, which is also found in literature as a synonymous for “social freezing”, puts the focus on the idea that oocyte cryopreservation for healthy women resembles other instances of elective medical interventions - such as cosmetic surgery - which generally have psychological benefits rather than therapeutic ones. This sparks the feeling that there is no reason why society should finance such desires of women who want to have children later in life. This is an interesting issue about social egg freezing: should the national healthcare system, where present, help women who desire postponing motherhood for non-medical reasons, by covering the cost of the procedures for these techniques? If oocyte cryopreservation is an accepted procedure to counter infertility and if fertility treatment is covered by public healthcare, should the logical consequence be that social freezing should also be covered by public national healthcare systems - or mandated insurance coverage - or that it should be admitted that there is a relevant distinction between ART with medical indications and ART with oocytes previously stored for non-medical reasons? (Borovecky et al., 2018). This aspect has also been debated in the literature and an objection to full coverage is that this could be a suboptimal allocation of scarce funds: healthcare budgets are strained and several countries are already struggling to accommodate the requests for ART for medical reasons and the added costs might be overwhelming (Mertes and Pennings 2012). Those who are in favour of a public coverage of social egg freezing costs base their belief on liberal ideology promoting “individual autonomy” exercised through informed consent, supporting the idea that a relational approach to autonomy may be a more suitable model for considering women’s choices about social egg freezing (Mutcherson, 2017; Shkedi-Rafid and Hashiloni-Dolev 2012).

Oocyte donation has also become an integral part of ART procedures as an alternative to embryo cryopreservation, which may not be an option for all couples who attend ART, because of personal religious or moral objections, or restrictive legislation in certain countries. In recent years, the demand for oocyte donation has increased, as it has become a treatment option for large numbers of women experiencing age-related infertility. Oocyte cryopreservation has led to the development of donor oocyte banks (Argyle et al. 2016). The cryopreservation of oocytes may have an impact on the number of available donor oocytes due to the fact that a certain number of women for whom oocytes were cryopreserved (both for medical and/or social reasons) will eventually not use all or any of their cryostored oocytes and may decide to donate them.

Until now, little attention has been given, in Italy, to the level of understanding women have of the implications of ovarian aging for fertility and reproductive planning, or of the medical options available, while in other countries some studies have looked at women’s attitudes, opinions, and knowledge about different aspects of social oocyte freezing (Ikhena-Abel et al. 2017; Lallemant et al. 2016; Lewis et al. 2016; Milman et al. 2017; O’Brien et al. 2017; Stoop et al. 2011; Stoop et al. 2015; Tan et al. 2014). In particular, two studies present interesting results on the baseline knowledge of oocyte cryopreservation in Eurapean and United States populations: one study comparing attitudes among Danish and British women (Lallemant et al. 2016) and the other conducted on medical students at Northwestern University in Chicago (US) (Ikhena-Abel et al. 2017), in which a high percentage of respondents demonstrated a good knowledge about oocyte cryopreservation (89% and 99% respectively).

With the aim of investigating attitudes, knowledge and intentions concerning social oocyte freezing among young Italian women, we have conducted a survey on a sample of female university students. This is an appropriate target population to assess in terms of attitudes towards egg freezing because these young women are thinking about their future reproductive decisions as they prepare for a demanding professional career following university courses and because they have an age suitable for useful egg freezing and storage (early and mid-20s). In our research, we wanted to assess different aspects of this phenomenon through a questionnaire consisting of five sets of questions – for a total of sixteen questions - covering respondents’:

1. socio-demographic characteristics (age, education, relationship status);
2. basic knowledge about social oocyte freezing;
3. personal attitude towards childbearing and social egg freezing;
4. opinion on cost coverage of social egg freezing;
5. attitude towards oocyte donation (to a known couple/woman or biobank).

Our results have been compared with those of other European countries and the United States that have been described above.

## Materials and methods

Once we established the general aims of the study, a questionnaire was developed by the research team, using a similar structure that had previously been used in another study involving our university students (Tozzo et al. 2017) and considering previous similar research on women’s attitudes towards social freezing. The questionnaire was subsequently piloted on a small number (n=30) of young women (19-30 years old) of reproductive age belonging to medical, administrative and professional staff at our insitution, reflective of potential respondents, and questions were modified with suggested changes made as appropriate, with the help of an expert in the field of statistics and survey development.

Data were collected during one academic semester in 2018, and the responses of 930 women were analyzed.

**Fig. 1:**
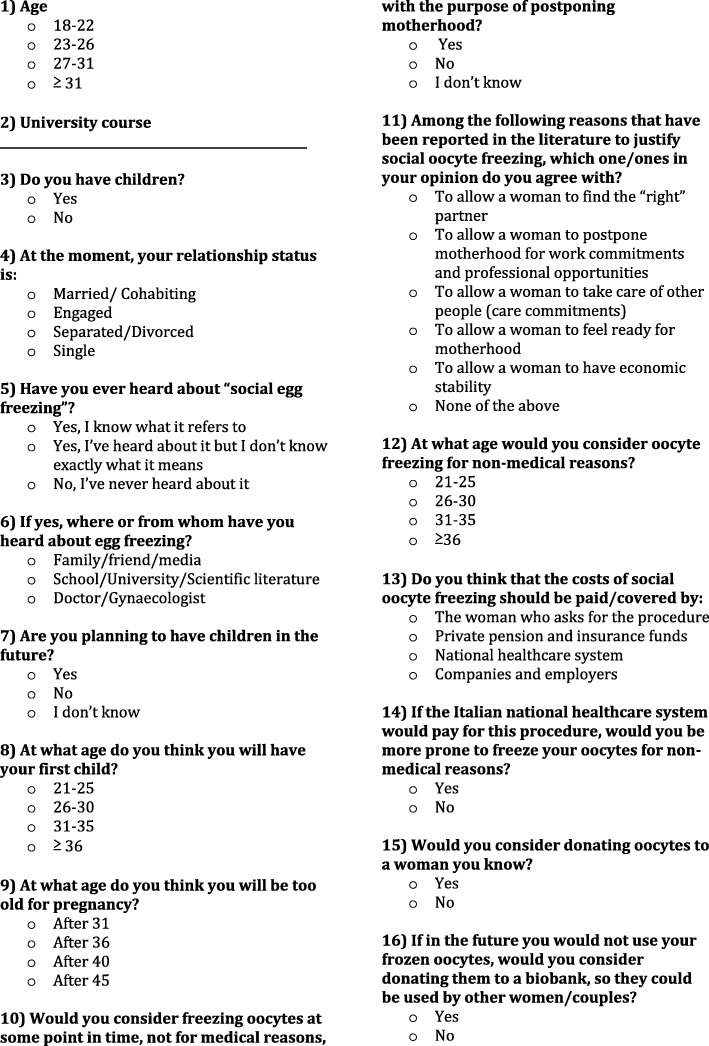
English translation of the questionnaire used in the study

A sample to which to administer the questionnaire was selected. We initially chose courses based on biomedical curricula and humanistic studies, but the questionnaire was administered only to students attending courses for which the president of each school had given specific authorization. In particular, healthcare professionals include: nurses, physiotherapists, speech therapists, health education professionals and midwives. A statistical analysis was conducted on the resulting data. Inclusion criteria for participation in the survey were female gender and current enrollment on university courses. All surveys were filled out anonymously (Fig. 1 contains the English translation of the questionnaire).

A printed-paper format was preferred for the questionnaire. It was drafted in Italian and distributed to female university students attending three different courses: law, medicine and healthcare professionals. This approach made it easy for respondents to answer the questions and consequently proved to be an effective way of obtaining a large number of completed questionnaires. At the end of their lectures, before the questionnaires were handed out, students were given a brief explanation of the purpose of the study (in the presence of the lecturer).

The questionnaire explored the socio-demographic characteristics of the respondents such as age, educational level, partnership status and previous children. Respondents were asked whether they had ever heard about oocyte cryopreservation for non-medical reasons, their source of information, and whether they would consider egg freezing for future use. For the latter, the response options were: “yes”, “no”, or “don’t know”. In order to obtain a better understanding of women’s attitudes towards egg freezing, participants were asked about possible non-medical justifications for egg freezing and at what age they think they would be more likely to freeze their eggs. Furthermore, we investigated age-related fertility decrease awareness, attitudes and intentions regarding the age for motherhood and child desire, attitudes towards oocyte donation (to both a biobank and a known woman or couple) and students’ opinions about public coverage of social egg freezing costs.

Data were inserted into an Excel file for statistical analysis using “STATISTICA” software (http://www.statsoft.com/Products/STATISTICA-Features). We initially used descriptive statistics, classifying and tabulating data in absolute and relative frequency tables.

The X^2^ test was used to analyze respondents’ willingness to freeze their eggs, their attitude towards the donating of their eggs to known/unknown women, and their opinion about who should pay for this procedure. The answers were then compared by relationship status, type of university course and knowledge about social egg freezing.

The competent Ethics Committee, consulted before starting the study, informed us that a specific authorization was not necessary but it was enough to communicate our intent to the presidents of the degree courses concerned and ask them for permission to submit the questionnaire to the students. All interested presidents expressed their favorable opinion.

## Results

Among the 950 questionnaires distributed, 930 were completed and returned. The respondents were medical students (32.5%), law students (34%) and healthcare-professional students (33.5%). Social and demographic characteristics of participants are reported in Table 1.

**Table 1:** Characteristics of survey participants

	*N*	%
Age, years		
18–22	744	80.0
23–26	134	14.4
27–31	26	2.8
≥ 31	17	1.8
University course		
Medicine	302	32.5
Law	316	34.0
Healthcare professionals	312	33.6
Relationship status		
Married/Cohabiting	28	3.0
Engaged	443	47.6
Single	453	48.7
No answer	6	0.6

The participants’ ages ranged from 18 to 35 even though the majority of respondents were 18-22 years old. 48.7% of them were single, 3% were married or cohabiting and 47.6% were engaged.

Twelve respondents (1.3%) already had children. 75.9% (n=706) wanted to have children in the future, with no differences among university courses. Concerning the assessment of when to time a pregnancy, 44.3% of respondents plan to become a mother at 31-35 years of age, 44.1% wish to have their first child at 26-30, while 73.4% would consider themselves too old for pregnancy after the age of 40. 62.2% of medical students plan to have their first child at 31-35 like 50.3% of law students, while the majority of healthcare students (64.7%) want to have their first child at 26-30.

Overall, 34.3% of students reported having heard of the possibility of oocyte cryopreservation for non-medical reasons and are aware of the meaning of this procedure; 23.8% have heard about this possibility but do not know exactly what this means and 41.7% have never heard about social egg freezing. In particular, 40.2% of law students and 32% of medical students have never heard about this procedure; the majority of healthcare-professional students (43.9%) have heard about this procedure but do not have a clear idea of the meaning of it. Among those who have already heard about social egg freezing, 34.2% of the respondents have heard about social egg freezing from family, friends and media, 22.5% reported learning about the procedure from school, university and scientific papers and only 1% from their doctor.

When asked whether they would consider freezing their eggs, 181 women (19.5%) responded “yes”, 363 (39.0%) “no” and 382 (41.0%) “don’t know”. There was a significant difference between different university courses with regard to the proportion of those reporting an intention to freeze their eggs. In particular, medical students seem more open to freezing their eggs (24.9%) than healthcare students (11.6%). The timing of uptake for social oocyte freezing was discussed. Most respondents (36.3%) felt that they may consider freezing their eggs at the age of 26-30. The majority of single (39%), married and cohabiting students (67.8%) answered “no” to the hypothesis of freezing their eggs, while the majority of engaged students (44.2%) responded “don’t know”.

Regarding the specific circumstances under which social oocyte freezing should be considered justifiable, students answered: “To allow a woman to find the “right” partner” and “To allow a woman to feel ready for motherhood” (26.5%), “To allow a woman to postpone motherhood for work commitments and professional opportunities” and “To allow a woman to have economic stability” (50.2%), “To allow a woman to take care of other people (care commitments)” (8.1%) and “None of the above” (14.1%).

With regard to the cost of oocyte freezing, 48.4% felt that payment for this elective procedure should be entirely paid by the woman herself, 33.5% think that payment should be covered by the national healthcare system, 13.9% by other forms of private insurance and 2.5% by companies and employers. However, should government subsidies be available, 50.0% (465 students) of the respondents may be more inclined to oocyte freezing. Nevertheless, the majority of healthcare students (54.7%) would not be more willing to freeze their oocytes even if the costs were covered by the national healthcare system.

When participants were asked if they would donate their oocytes to a woman they know, the majority of students (64.9%) would not accept donating, with no differences among different courses. When asked about the donation of oocytes to a biobank, this was considered an option by 42.5% of the respondents, in particular by 51.2% of medical students, by 34.5% of law students and by 44.2% of healthcare-professional students.

In Table 2, we have summarized the results regarding awareness of social egg freezing, willingness to freeze eggs in the future, and attitudes towards donation to a biobank, considering different university courses.

**Table 2:** Social egg freezing awareness, willingness to freeze and willingness to donate to a biobank by university course

	Medicine	Law	Healthcare Professionals
Question			
Have you ever heard about “social egg freezing”? (yes,%)	33.2	33.2	33.5
Would you consider freezing oocytes at some point in life, not for medical reasons, with the purpose of postponing motherhood? (yes,%)	24.9	22.2	11.6
If in the future, after deciding not to use your frozen oocytes, would you consider donating them to a biobank, so they could be used by other women/couples? (yes,%)	38.5	27.1	34.4

The results regarding attitudes towards donation are reported in Table 3.

**Table 3:** Attitudes of women towards oocyte donation

	Freezers	Doubtful	Non-freezers
Question			
Would you consider donating oocyte to someone you know? (yes,%)	44.1	38.4	24.2
Would you consider anonymously donating oocyte to a biobank (yes,%)	55.0	51.5	28.6

## Discussion

To our knowledge, this is the first study to examine Italian young female awarness on age-related infertility and their attitudes regarding acceptable indications for elective oocyte freezing, their potential personal use, in what circumstances cryopreserved eggs should then be used and their attitudes towards cost coverage and oocyte donation.

The study population is represented by 930 young female students at the University of Padova, Italy, mostly between the ages of 18 and 22.

In our research, many students (41.7%) had never heard about social egg freezing while 34.3% were aware of this phenomenon, thus documenting that, in Italy, young women, even if well-educated and despite their university choice, are not aware of this opportunity (see Table 2). It is interesting to underline that only 1% of respondents had heard about social egg freezing from their doctors while the majority (34.2%) had gained information on this topic from families, media and friends, so the information they had may have been inaccurate, partial, transmitted through conventional wisdom and likely not based on scientific assumptions. This data could suggest that, normally, treating physicians, especially gynecologists, provide little information on this issue and accurate information on the details regarding social oocyte freezing should be made more available to the public for it to be a viable option (Bracewell-Milnes et al. 2018; Hvidman et al. 2015; Lemoine and Ravitsky 2015). While not a direct comparison due to different survey styles, baseline knowledge of oocyte cryopreservation in our population was similar to that reported by Singaporean medical students (36.4%) (Tan et al. 2014) and was lower than that reported in a study comparing attitudes between Danish and British women in 2012 (Lallemant et al. 2016) and about medical students at Northwestern University in Chicago (US) (Ikhena-Abel et al. 2017), in which, respectively, 89% and 99% of respondents had heard about oocyte cryopreservation.

The findings of this study, in terms of willingness to freeze eggs, concur with other surveys assessing acceptance of elective oocyte freezing in other countries: we found that only 181 women (19.5%) would accept freezing their eggs for social reasons, and this percentage is similar to that reported in a US research study published by Milman et al. (2017) (21.6%), in the study comparing attitudes of Danish and British women (19%), and in the Singaporean study (26.4%) and slighty lower than the number of “potential egg freezers” reported in a previous Belgian study by Stoop et al. (2011) (31.5%). Considering medical students, only 24.9% consider themselves to be potential future freezers and this percentage is lower than the results seen in the surveys of Singaporean (48.9%) and American medical students (71%). In our study, there was a significant difference between different university courses with regard to proportions reporting the intention to freeze eggs, in particular between medical students and healthcare students: actually 24.9% of medical students think of using social egg freezing in the future and only the 11.6% of healthcare-professional students answered “yes” to the same question (see Table 2).

Considering the relationship status, the majority of single (39.1%), married and cohabiting (67.9%) students responded “no” to the hypothesis of freezing their eggs while the majority of engaged (44.2%) students responded “don’t know”. Among single students, 39.1% responded “no” to the hypothesis of egg freezing and 22.1% answered “yes”. This may indicate that the question was referring to women’s general attitude with regard to egg freezing rather than to the respondents’ relationship status.

The majority of respondents consider it to be too late to have children after 40 years of age, while only 23.9% answered “after 36 years of age”, which seems to be a more correct answer considering the natural decline in women’s fertility after 35 years of age (Jackson 2016; Lewis et al. 2016). In the study performed by Ikhena-Abel et al. (2017) on medical students at a US university, 72% of respondents correctly identified the age at which fertility significantly declines as age 35. Surprisingly, in our survey, in addition to underestimating the decline in fertility between 36 and 40 years, what seems to be unknown is the age at which egg freezing should be performed: in fact, 79.6% of students think they are too old for pregnancy after 40 and 71.72% of all respondents think that the better age to freeze their oocytes is after 26, rather than the interval 21-25 years of age which is the more appropriate one, considering what has been reported in literature as the optimum age to freeze oocytes (Jackson 2016; Stoop et al. 2014). Given that the optimum time to freeze one’s eggs, from a clinical point of view, would be during a woman’s 20s (Jackson 2016), we can argue that in our surveyed population there is a large gap in awareness about true technical aspects of social freezing, so it may be important to raise young women’s awareness on improved embryo quality and the reduced prevalence of aneuploidy oocytes at a younger age, keeping in mind that the majority of our respondents (44.3%) planned to have their first child between the ages of 31 and 35 years old.

Interestingly, regarding the reasons underlying the choice to freeze eggs, female students at our university seem to consider economic reasons as the more important in justifying social egg freezing. When analyzing the willingness to freeze oocytes and their relationship status, it seems that being engaged or single does not really affect the potential choice to freeze. Furthermore, among single students, the majority of them think that economic and professional reasons are more important in justifying the choice to freeze eggs. These data suggest that the “emotional” aspects have little influence on the opinion of our female students on the possibility of freezing or not freezing their oocytes for non-medical reasons.

Egg freezing for fertility preservation in non-medical situations is generally paid out-of-pocket by the woman, but the costs of undergoing oocyte cryopreservation become an important factor for the individual who may decide to use this technique and an important factor for society and other potential payers to be considered (Mertes and Pennings 2012), so in this paper we have also addressed the question of cost coverage. We found that most respondents (48.4%) felt that the payment for this elective procedure should be entirely borne by the patient, while 33.5% were in favour of public coverage. We have noticed that students are scarcely aware of the possibility that employers and companies could offer this procedure as a benefit to their female workers: in fact, only 2.5% of students think that employers and companies should pay for social egg freezing. However, should government subsidies be available, 50.0% (465 students) of the respondents may be more inclined to oocyte freezing. In particular, medical students would tend to consider more the possibility of freezing their eggs if the payment was covered by the national healthcare system: in fact, the percentage of those, among medical students, who would do social freezing rises from 24.9% to 56.4% in the case of public cost coverage. On the contrary, the majority of healthcare-professional students (54.7%) would not be more willing to freeze their oocytes even if the costs were covered by the national healthcare system. One possible explanation for this difference is that medical students are more aware that their study path before entering the world of work and enjoying stability will be very long and then, in the presence of funding from the state, they tend to consider social freezing as a good option for delaying motherhood. Furthermore, 47.51% of respondents who would consider social egg freezing in the future are those who believe that the cost should be covered by the state. On the contrary, most of those who do not think of social freezing for themselves (64.8%) believe that costs should be entirely paid by the woman. In other words, those who do not resort to social freezing for themselves still consider it a woman’s choice that should not be economically supported by the state. It is noteworthy that the majority of respondents that think that reasons related to their relationship status may justify social egg freezing also think that the costs should be paid by the woman herself, while respondents believing that economic reasons may justify social egg freezing consider that the costs should be publicly covered. It seems that those who responded to our questionnaire believe that women who choose egg freezing for economic or career reasons should be economically supported by the community, while those who choose social freezing for reasons related to relationship status should pay for it, probably because these reasons are considered by our sample less important than the economic ones and therefore less worthy of a public contribution.

When considering egg donation, in general, female students at our university are not willing to donate their eggs and there is a greater tendency to donate to a biobank (42.5%) rather than to women or couples they know well (33.4%); law students are those who would tend to donate less, probably because they are more aware, and probably worried about the possible juridical, and perhaps ethical, implications of gamete donation. Pure results in the present survey have confirmed data collected in a previous survey conducted on university students about knowledge, awareness and attitudes towards biobanks. Specifically, also in that case, we found that law students were less open to donating their biological samples to a biobank than medical students (Tozzo et al. 2017).

It is interesting to underline that respondents more willing to freeze their eggs are also those more inclined to donate to a biobank (see Table 3). This study also shows that women who are potentially interested in social oocyte freezing are also more open to the idea of donating oocytes. In order to explain the attitude of our surveyed women towards egg donation, we have to take into account that potential donors need to overcome twin hurdles: the first with regard to the physically demanding procedures of ovarian stimulation and oocyte retrieval; secondly, with regard to the psychological burden of becoming the genetic parent of a child that one may know, in case of donation to a known woman/couple, or may not know in case of donation to a biobank (Mertes et al. 2012). When donation to a friend or family member is considered, many, if not most, women of our sample may have found the idea of having a genetic child growing up in a known family could be emotionally troubling, and, therefore, even if the physical burden is lifted, it is unlikely that large numbers of women with spare oocytes will be donating them for reproductive purposes. Unlike other areas in which the culture of donation is strongly rooted in the Italian female population (Italian Government. Health Ministry 2019), for example organ donation (Canova et al. 2006) or samples donation for research (Tozzo et al. 2017), and in which motivation is linked to the awareness of the great ethical value of organ donation and to a widespread sentiment of civic altruism and human solidarity, the idea of gamete donation finds resistance probably because of particular cultural and religious convictions and of a particular sensitivity about certain ethical issues. This propensity shows that there may be weak motivations, ethical or spiritual, which support the choice of gamete donation.

Although our questionnaire was administered to a particular sample of well-educated young Italian women, who cannot represent the Italian population at large, our findings suggest that young Italian women are significantly less aware of age-related decline in fertility and the possibility of using social egg freezing compared with their similarly situated counterparts in other Western countries. In general, the tendency seems to be to justify this procedure more for economic reasons than for those related to relationship status and there is a tendency to consider such procedures not worthy of economic support from the state. On the theme of gamete donation, in our sample we found a tendency not to donate, especially to known people.

Limitations of this study include a single university being studied and a study population limited only to university students, who represent a more knowledgeable group of women than the general population, so generalization could be questionable. Nevertheless, we believe that our study population, since these students will eventually start some of the most demanding careers for women, is an interesting target population to understand how young Italian women who are planning a professional career are approaching their reproductive life. Furthermore, the study population is of reproductive-aged women for whom the question is most pertinent considering the best age to freeze oocytes. Further research with the aim of verifying these single-university findings and to improve the general applicability of the results is required, along with more research to clarify the reasons for the refusal to engage in oocyte donation.

## Conclusions

In conclusion, data collected in this study revealed some important elements about social oocyte freezing in Italy. In particular, they emphasize the fact that questions on social freezing are new and timely in our country and the medical community should be involved in debating and answering these questions in order to give young patients correct information about both fertility and technical possibilities to preserve it and, eventually, defer childbearing.

In our country, it is certainly necessary to raise a greater awareness about fertility issues, both in the female population and in the medical and scientific community, so as to encourage health professionals to better inform their patients.

More information on fertility issues and possible remedies to age-related infertility is not only useful for postponing parenting, but it can also offer a more concrete and conscious reproductive autonomy, which is desirable to be realized independently of career pressures and lack of services in favour of responsible parenting. However, this information process has to also take into account other equally important concerns regarding the possibility of creating high and potentially false hopes and introducing medical processes to primary fertile women.

## Abbreviations

ART: Assisted Reproductive Technology
ICSI: Intracytoplasmic Sperm Injection
IVF: In Vitro Fertilization
